# Continuous exchange of nectar nutrients in an Oriental hornet colony

**DOI:** 10.1038/s42003-022-04095-0

**Published:** 2022-10-20

**Authors:** Sofia Bouchebti, Levona Bodner, Eran Levin

**Affiliations:** School of Zoology, George S. Wise Faculty of Life Sciences, Tel Aviv University, Tel Aviv, 6997801 Israel

**Keywords:** Ecology, Entomology, Stable isotope analysis

## Abstract

Nutritional exchanges play a fundamental role in the evolution of animal societies. In higher animal societies, while adult individuals can be both food donors and receivers, the offspring usually only receive food from the adults. Hornets and wasps are fierce insect hunters that feed their larvae with prey. However, although the adults also consume floral nectar, the role of nectar in vespid nutrition has remained largely unknown. We provided experimental colonies of the Oriental hornet with artificial nectar enriched with a ^13^C-labeled amino acid, and found that a continuous cycle of nutrition took place, in which nectar nutrients were used and exchanged back and forth between adults and larvae. We posit that this continuous cycle of nutrients constitutes a mechanism contributing to social cohesion. In an additional experiment, we found that nectar consumption was essential for adult and larval survival, suggesting the importance of wasps and hornets as pollinators in natural ecosystems.

## Abstract (web summary)

Amino acid tracing reveals a unique nutrient exchange system in Oriental hornets, with continuous exchange of nectar nutrients across all life stages.

## Introduction

Nourishment of offspring is one of the first evolutionary steps leading to sociality^1^. In complex animal societies, such as killer whales, vampire bats, and primates, reciprocal feeding can also take place between adult individuals of the same group^2–4^. In eusocial insects, feeding reaches a further level of complexity since food items are usually collected by only a small number of individuals (foragers) and then disseminated among all members of the colony^5^. This social feeding unites all colony members with one another^6^.

Nutrient exchange among adult individuals in social insects is bidirectional, i.e., they can be both donors and receivers of the same type of food or nutrient^7,8^. In contrast, for the larvae, this exchange is generally unidirectional, as larvae are sessile and must receive their food from adults^6^. In some species of hymenopterans, however, the larvae secrete a liquid back to the adults that facilitates food-processing by the adults^9,10^. In the fire ant *Solenopsis invicta* and the leaf-cutter ant *Acromyrmex subterraneus*, the larvae possess a higher and more diverse digestive enzymatic profile than that of the adults^11,12^. It has been suggested that these larval secretions contain digestive enzymes that promote food processing when consumed by adults^9,11,13^. In social wasps, larval secretions constitute one of the primary adult food resources^7,14–16^. Wasp larvae are fed with prey (arthropod or carrion) collected by adults and, in exchange, the larvae deliver back to the adults the protein degradation products as a secretion containing simple carbohydrates and free amino acids (resulting from gluconeogenesis and protein breakdown, respectively)^14,17^. Although wasp workers can partially digest the proteins obtained from their prey, males are unable to do so and rely entirely on larvae for protein digestion^10^. This both reciprocal and unilateral exchange of nutrients (i.e., protein versus carbohydrates and free amino acids), and consequent nutritional co-dependency between adults and larvae, is suggested to be key to establishing sociality in Vespidae^10,15^.

Despite social wasps being fierce insect hunters, adults are also often observed feeding on floral nectar from a variety of different plant species^18^. The Asian hornet (*Vespa velutina nigrithorax)*, for instance, has been observed feeding on floral nectar of 27 plant species^19^. This nectar consumption, however, has often been characterized simply as an opportunistic behavior, as flower-visiting can also be associated with prey hunting^19^. Adult workers are strong flyers with an extremely high metabolic rate^20^, and fuel their metabolism with a high carbohydrate and free amino acids diet found in nectar and larval secretions^15,21^. Such carbohydrate-rich diets might also protect their flight muscles from oxidative stress derived from their high aerobic performance^22^. The macronutrient composition of larval secretions is analogous to the nectar of plants visited by vespids, although it is generally richer in its amino acid concentration and diversity^23^. In contrast to workers, larvae require amino-acid-rich food in order to synthesize the proteins necessary for their development, and they are sustained on a highly proteinaceous diet. Since larvae are fed with prey, and larval secretions are more nutritious for the adults than nectar, why do these wasps also forage on floral nectar? Hunt et al. reported that the larvae of *Polybia occidentalis* received some of the nectar collected by the adults, and thus that nectar could potentially represent an additional resource for the larvae^24^. However, the role of nectar in wasp nutrition has remained largely unknown.

Nectar collected by social insects is typically stored in the crop and transferred to other individuals via a mouth-to-mouth exchange known as trophallaxis^9^. Trophallaxis is an efficient mechanism for the rapid transfer of nutrients among all colony members^24–27^. To date, food dyes^28,29^, radioactive-labeled food^24,26,30–33^, and, recently, fluorescent-dyed food^34,35^, have been used to study the path of resources transferred via trophallaxis in hymenopterans. While these methods have provided valuable information on the food exchange dynamics within the colony, they are limited by the constraints of labeling the food resources as a whole. In contrast, using stable isotopically-labeled macronutrients enables the study of how a specific nutrient, or even a specific carbon, present in the food resource is transferred, allocated, and utilized by each colony member^10,36^.

In this study, we used artificial nectar enriched with a ^13^C_1_ essential ketogenic amino acid (leucine) to investigate how a specific macronutrient in nectar is utilized and exchanged among the colony of a social vespid, the Oriental hornet (*Vespa orientalis*). We also investigated the importance of nectar for the survival of the colony. Our results reveal a major role of nectar in hornet nutrition. We show that nectar nutrients are exchanged in a continuous cycle among all the different life stages of the colony members, challenging the prevailing knowledge in this field to date.

## Results

### Experiment 1: Transfer and allocation of a nectar macronutrient into body tissues

Six experimental colonies containing ten larvae and three workers were fed with artificial nectar enriched with labeled leucine for seven days (six control experimental colonies were fed with unlabeled artificial nectar).

**Fig. 1:**
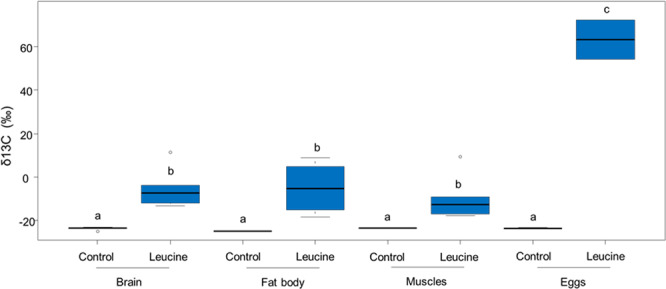
Allocation of 13C-leucine into adult tissues. δ^13^C values (‰) in the body tissues (brain: *N* = 12; fat body: *N* = 8; and muscles: *N* = 10) and eggs (*N* = 6) of adult hornets fed with ^13^C-leucine-enriched artificial nectar (in blue), and their controls (red). A higher delta indicates higher ^13^C levels. The boxes represent the first and third quartiles and the median. The whiskers represent the maximum and minimum values. The circles represent the outliers. A generalized linear mixed model followed by post-hoc pairwise comparisons (Tukey adjusted) was used for statistical analysis. Different letters indicate a pairwise-comparison with *P* < 0.05.

**Fig. 2:**
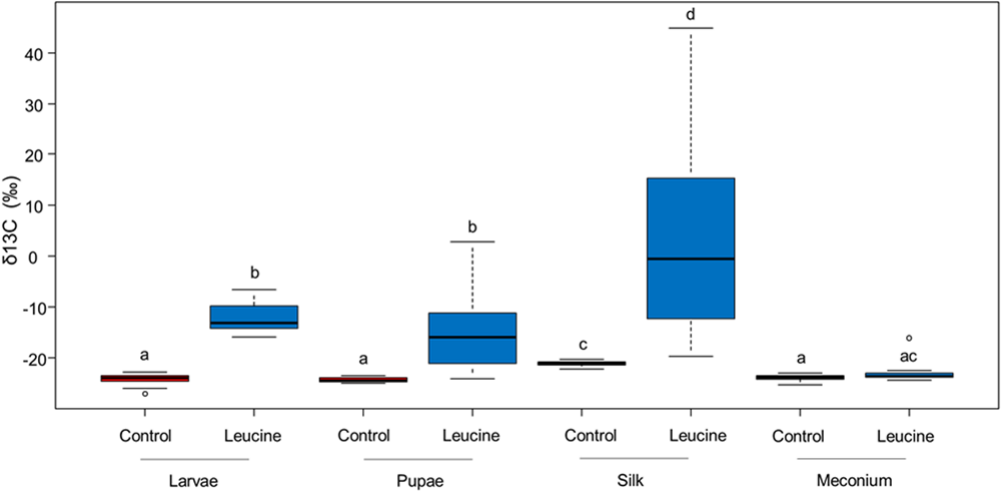
Allocation of 13C-leucine into brood tissues. δ^13^C values (‰) in larvae (*N* = 42), pupae (*N* = 21), silk (*N* = 19), and meconium (*N* = 29) from colonies fed with ^13^C-leucine-enriched artificial nectar (in blue), and their controls (in red). A higher delta indicates higher ^13^C levels. The boxes represent the first and third quartiles and the median. The whiskers represent the maximum and minimum values. The circles represent the outliers. A generalized linear mixed model followed by post-hoc pairwise comparisons (Tukey adjusted) was used for statistical analysis. Different letters indicate a pairwise-comparison with *P* < 0.05.

Labeled carbons from leucine were assimilated into adult hornet body tissues and eggs, with a higher assimilation in the eggs (Fig. 1; treatment: F_1,27.300_ = 330.485, *P* < 0.0001; body tissue: F_3,27.638_ = 9.818, *P* = 0.0001; treatment × body tissue: F_3,27.638_ = 7.284, *P* = 0.0009). However, the workers also used nectar to feed the larvae. Labeled leucine was found in the body tissues of the larvae, the pupae, and in the silk, but not in the meconium (Fig. 2, treatment: F_1,67.431_ = 129.075, *P* < 0.0001; body tissue: F_3,92.093_ = 36.990, *P* < 0.0001; treatment × body tissue: F_3,94.961_ = 16.737, *P* < 0.0001).

### Experiment 2: Allocation of a nectar and a prey macronutrient in the larval secretions

Two experimental colonies containing 100 larvae and 30 workers were fed for seven days with either artificial nectar enriched with labeled leucine and unlabeled prey items (bumble bees), unlabeled artificial nectar and protein-labeled prey item (labeled leucine), or unlabeled artificial nectar and unlabeled prey items (control).

**Fig. 3:**
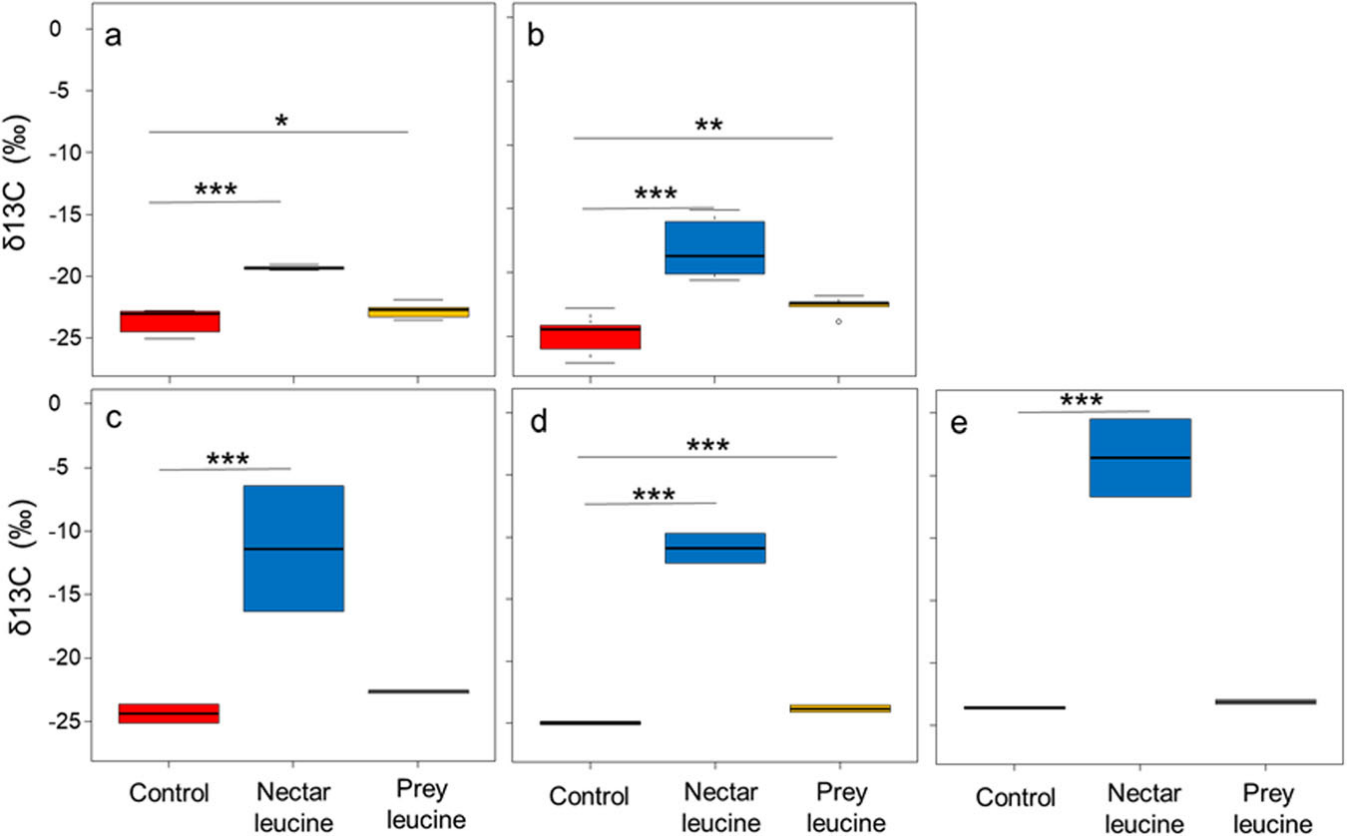
Allocation of 13C-leucine into adult and larval tissues. δ^13^C values (‰) in larval samples (**a** larval secretions (*N* = 26) and **b** larval bodies (*N* = 18)) and adult samples (**c** brain (*N* = 6), **d** fat body (*N* = 6), and **e** muscles (*N* = 6)) from colonies fed with either ^13^C-leucine-enriched nectar (in blue) or ^13^C-leucine-labeled bumble bees (in yellow), and their control (in red). A higher delta indicates higher ^13^C levels. The boxes represent the first and third quartiles and the median. The whiskers represent the maximum and minimum values. Kruskal-Wallis rank sum test and generalized linear mixed models followed by Dunnett’s multiple comparisons with control were used for statistical analysis. *P ≤ 0.05, **P ≤ 0.01, ***P ≤ 0.001.

The labeled carbons of leucine originated from artificial nectar and prey items were found in the larval secretions (Fig. 3, χ^2^ = 17.273, DF = 2, *P* = 0.0001). This labeled macronutrient was also found in the larval body tissue (F_2,14_ = 18.575, *P* = 0.0001, Dunnett’s Multiple Comparisons: control versus nectar leucine, *z* = 6.092, *P* < 0.0001, control versus prey leucine, z = 3.210, *P* = 0.002), and in most of the adults’ tissues (brain: F_2,2_ = 28.844, *P* < 0.033, Dunnett’s Multiple Comparisons: control versus nectar leucine, z = 7.347, *P* < 0.0001, control versus prey leucine, z = 2.004, *P* = 0.082; fat body: F_2,2_ = 2264.500, *P* = 0.0004, Dunnett’s Multiple Comparisons: control versus nectar leucine, *z* = 63.17, *P* < 0.0001, control versus prey leucine, *z* = 11.480, *P* < 0.0001; muscles: F_2,3_ = 149.47, *P* = 0.0009, Dunnett’s Multiple Comparisons: control versus nectar leucine, *z* = 15.433, *P* < 0.0001, control versus prey leucine, *z* = 0.967, *P* = 0.524), confirming that both larvae and adults consumed nectar and prey.

### Experiment 3: Effect of nectar on survival

Thirty experimental colonies containing ten larvae and three workers were fed for one week with either a nectar-only diet (unlabeled artificial nectar), a nectar and prey diet (unlabeled artificial nectar and unlabeled bumble bees), or a prey-only diet (unlabeled bumble bees).

**Fig. 4:**
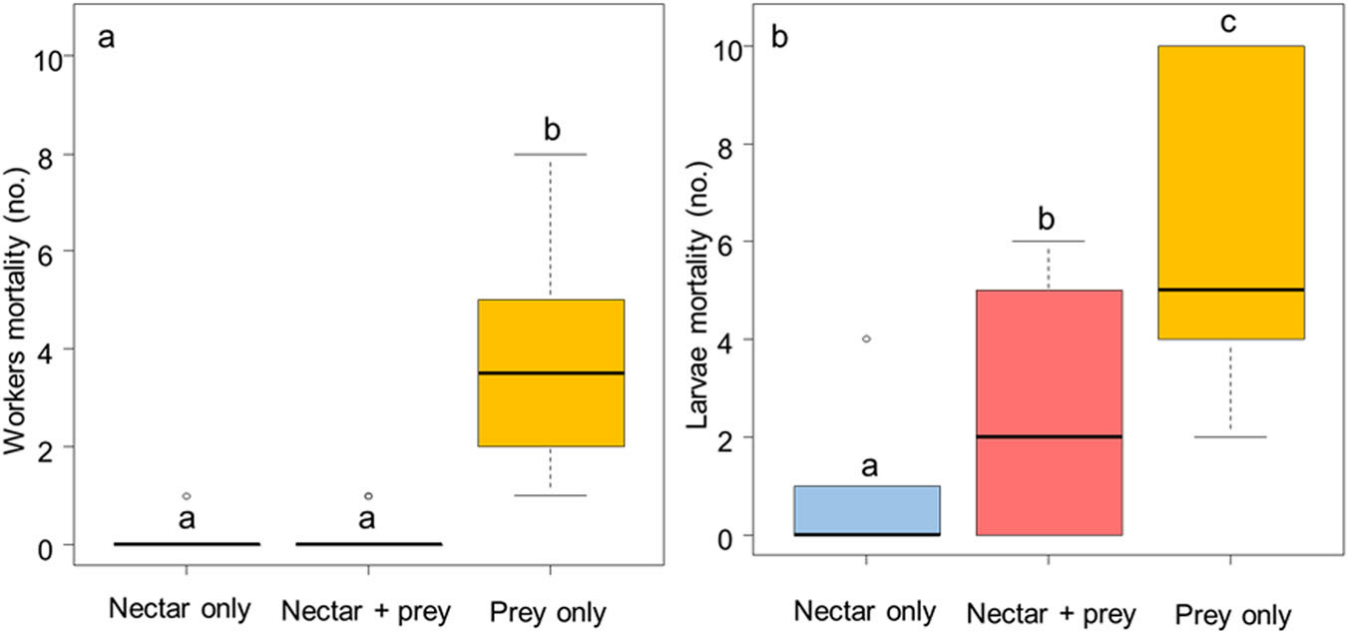
Mortality of hornets fed with different diets. Mortality of workers **a** and larvae **b** fed with nectar only (in light blue), nectar and prey (in light red), and prey only (in yellow) (workers; nectar only group: *N* = 31, nectar + prey group: *N* = 32, and prey only group: *N* = 68; larvae: *N* = 100 per group). The boxes represent the first and third quartiles and the median. The whiskers represent the maximum and minimum values. The circles represent the outliers. A generalized linear mixed model followed by post-hoc pairwise comparisons (Tukey adjusted) was used for statistical analysis. Different letters indicate a pairwise-comparison with *P* < 0.05.

Adult hornets could not survive without nectar in their diet (Fig. 4A, F_2,27.758_ = 13.879, *P* < 0.0001). Larval mortality also dramatically increased without nectar (Fig. 4 B, F_2,39.471_ = 19.735, *P* < 0.0001).

## Discussion

The “social wasp nutritional theory” suggests a nutritional co-dependency between adults and larvae, with adults feeding the larvae with protein only and larvae feeding back the adults with carbohydrates and free amino acids^15^. Here we counter this theory and demonstrate that a same nutrient can be used and exchanged between the different life stages in a continuous cycle: from the adults to the larvae and back. We found that a nectar-derived macronutrient, the essential amino acid leucine, was allocated to both adult and larval tissues and exchanged back and forth via trophallaxis. We also found that floral nectar is critical for the colony’s survival: neither adults nor larvae were able to survive without this additional nutritional source. Our study thus reveals that: (1) adult wasps do not nutritionally depend on the larvae but can acquire amino acids directly from the nectar; and (2) prey are not the only food resource for larvae, which can also be fed simple nectar-derived nutrients such as amino acids.

It was previously found that worker hornets use nectar-derived nutrients to fuel their metabolism^21,37^. Here we have shown that the nectar-derived leucine was incorporated into all the examined adult body tissues (brain, fat body, and muscles) as well as into the eggs laid by the workers (in the absence of a queen, workers develop ovaries and lay unfertilized eggs^38^). Our results corroborate the findings that in social vespids, such as the Oriental hornet, nectar-derived nutrients are used for reproductive and somatic needs, as previously demonstrated in lepidopterans^39^. However, unlike non-social insects, and similar to bees^40^, worker hornets also use a portion of the consumed nectar to nourish their larvae. The larvae use these nectar-derived nutrients for both development and silk production, as labeled leucine was found to be assimilated in larval and pupal body tissues and in the silk caps of the pupae. We found no traces of labeled leucine in the meconium of the pupated larvae, suggesting that no nutrient remains unused during larval development. Our findings thereby confirm those of Hunt et al., demonstrating that larvae can also be fed with the nectar collected by foragers^24^. Surprisingly, we found that larvae transfer back to the workers some of the nutrients that they had previously received from them. These findings indicate that the nutrients in larval secretions do not exclusively originate from gluconeogenesis and protein breakdown, refuting previous suggestions^14,17^. However, further experiments are needed to quantitatively assess the origin of each type of food (prey versus nectar) in the larval secretion composition. Our results support the nutritional theory that reciprocal feeding between adults and larvae has constituted an important driving force in the evolution of sociality in wasps^15^. However, we suggest that a continuous cyclic exchange of nutrients among all colony members, rather than a unilateral exchange of specific nutrients (i.e., protein versus free amino acids and carbohydrates), is a more probable mechanism involved in social cohesion.

Although social wasps are known to consume floral nectar, their role as pollinators in ecosystems is often overlooked (but see^18^ for a review). In the scientific literature, the reported larval nutritional need for protein has completely overshadowed the discussion of the need for additional nutrients^15^. Here, we have shown that neither larval secretions alone nor prey alone were sufficient to sustain the nutritional needs of adults and larvae, respectively, and that without the supply of an additional carbohydrate source, the mortality of both workers and larvae increased dramatically. Foraging on flowers is thus not facultative for social wasps; and, similarly to bees, wasps too should be considered as important pollinators. Honey bee larvae, for example, require a percentage of carbohydrates ranging from 14.58 to 26.30% in order to develop correctly^41^. It is not surprising, therefore, that hornet larvae require an additional source of carbohydrates, such as nectar, in their diet. Adults of social Vespidae species are also often observed to consume ripe fruits^15^, although whether they feed their larvae with fruits remains to be investigated. Surprisingly, a diet comprising both nectar and prey led to a higher larval mortality than a diet comprising nectar alone. This may have been due to the fact that in our experiment the concentrations of amino acids and sucrose in the artificial nectar were fixed, and the hornets were unable to balance their nutrient intake. High concentrations of dietary amino acids are toxic and induce high mortality in many social insects^42–45^. However, it is important to note that the content of amino acids in nectar is much lower than that in the insect prey^46,47^. Thus, although the survival of larvae was not affected by a nectar-only diet during our seven-day experiment, it is unlikely that larvae can fully develop without also a supply of prey.

Floral nectar composition and concentration vary considerably within and between species^48^. The artificial nectar used in our study, consisting of a 50% sucrose solution and a mix of 10 essential amino acids, probably differs from the nectar naturally collected by the Oriental hornet. Further studies characterizing the plant species and composition of nectar consumed and collected by the Oriental hornet under natural conditions are necessary in order to obtain a better understanding of the role of nectar in hornet nutrition.

Using a ^13^C labeled nutrient, we revealed a nutritional cycle that occurs via trophallaxis in a social insect. Trophallaxis also takes place in other social insects and mammals^2,9,49–51^. Consequently, studying the utilization and exchange of an individual nutrient within a social group will provide a deeper understanding of the role of nutritional interactions in shaping sociality.

## Methods

### Insect collection and maintenance

The Oriental hornet (*Vespa orientalis)* is a large, eusocial wasp, mostly found in south-eastern Europe, North Africa, and western Asia, and is currently spreading worldwide^52^. This species establishes annual colonies in underground cavities, comprising up to several thousand individuals^53^.

Six colonies of Oriental hornets collected from areas around Tel Aviv University were used to create our experimental colonies (2 to 15, depending on the experiment). The combs, containing eggs, larvae, and pupae, were excavated from the ground and adult workers were collected from the excavated colonies using a sweeping net. Experimental colonies were created within one hour following the excavations. The experiments were conducted in a climate-controlled room (25 ± 2 °C, 75 ± 10% RH). We used bumble bees (*Bombus terrestris*), a common protein source for hornets^10,54^, to feed the experimental colonies, according to our experimental design. The bumble bee colony was purchased from AgroBee, Ein Yahav, Israel.

### Experiment 1: Transfer and allocation of a nectar macronutrient into body tissues

To determine how a single nectar macronutrient is transferred and allocated into the body tissues of individuals at different life stages, we created experimental colonies comprising pieces of comb containing ten larvae of 1st to 5th instars, and glued the combs to the ceiling of wooden boxes (10 × 14.4 × 12 cm), each with three of their respective workers.

Each experimental colony was supplied ad libitum with a test tube containing a 50% sucrose solution and an additional water tube. Two dead adult bumble bees were provided every second day as a protein source.

The sucrose solution was enriched with 1 mM of ^13^C_1_-labeled leucine (Cambridge Isotope Laboratories, Tewksbury, MA, USA) in the treatment groups, while control groups were provided with sucrose solution only. Six replicates (i.e., experimental colonies) were carried out per group.

After seven days, the experimental colonies were sacrificed by freezing, and the different tissues were dissected out, thoroughly washed with distilled water, and dried at 60 °C for three days. The brain, fat body, and muscle tissues of adult hornets, and the body tissues of the larvae, were collected for analysis. During the seven days of treatment, some adult hornets laid eggs and some larvae pupated. The eggs and the pupae’s body tissues, silks, and meconium (i.e., larval feces discharged before pupation) were therefore also collected and analyzed.

### Experiment 2: Allocation of a nectar and prey macronutrient into larval secretions

In this experiment, we investigated the origin (nectar and/or prey) of the nutrients present in larval secretions. To collect a sufficient amount of secretion for the isotope analyses, we used larger experimental colonies: pieces of combs containing around 100 larvae were placed in large wooden boxes (14 cubic liters), along with 30 of their respective workers. Each experimental colony was supplied ad libitum with a test tube containing a 50% sucrose solution and an additional water tube. Ten dead adult bumble bees were provided every second day as a protein source. Three different diets were used in this experiment. The first treatment group was provided with a sucrose solution containing 1 mM of ^13^C_1_- leucine and unlabeled bumble bees. The second treatment group was provided with unlabeled sucrose solution and protein-labeled bumble bees fed with ^13^C_1_-leucine enriched sucrose solution (100 mg ^13^C_1_-leucine per 25 ml of 50% sucrose solution) for seven days and killed by freezing (as described in ref. 10). The control group was provided with unlabeled sucrose solution and unlabeled bumble bees.

After seven days, the adult hornets were anesthetized with diethyl ether and removed from the boxes. Larval secretions were then collected with a capillary pipette by gently stimulating the mandibular region of the larvae. To verify that the labeled nutrients had been fully ingested and assimilated into the hornet bodies, tissues of two workers (brain, fat body, and muscles) and three larvae per replicate were analyzed for δ^13^C levels. The larval secretions and tissue samples were dried at 60 °C for three days prior to analysis.

Two replicates (i.e., experimental colonies) were carried out per group, and three to five larval secretion samples per replicate were analyzed.

### Experiment 3: Effect of nectar on survival

To assess the importance of nectar in hornet nutrition, we used experimental colonies comprising pieces of comb containing ten 1st–5th instar larvae glued to the ceiling of wooden boxes (10 × 14.4 × 12 cm) with three of their respective workers. Each experimental colony was supplied ad libitum with a tube of water. Three different diets were used in this experiment. The first group (nectar-only diet) was provided ad libitum with artificial nectar composed of 50% sucrose solution mixed with the following ten essential amino acids at equimolar concentrations (1 mM): methionine, tryptophan, leucine, lysine, valine, arginine, isoleucine, phenylalanine, threonine, and histidine (Sigma-Aldrich, Israel). The second group (prey-only diet) was provided with dead adult bumble bees only (two bumble bees every second day). The third group (nectar and prey diet) was provided with artificial nectar (as used in the nectar-only diet group) and dead adult bumble bees (as used in the prey-only diet group). Dead adult workers were counted daily and removed from the boxes. After seven days, adult hornets were anesthetized with diethyl ether and removed from the boxes, and the number of live larvae in the combs was counted. To avoid biasing the mortality of the larvae with the mortality of the adults, dead adults were immediately replaced with new ones from their respective colony. Ten replicates (i.e., experimental colonies) were carried out per group.

### Stable isotope analyses

Samples of 1 mg of each dry tissue (larval body tissue, pupal body tissue, silk, meconium, adult brain tissue, adult fat body tissue, adult muscles tissue, eggs, and larval secretions) were loaded into tin capsules. For egg analyses, in order to reach the 1 mg minimum of dry mass, we pooled several (five to 11) eggs from each replicate. The δ^13^C (‰) values in the samples were measured and calculated using a Picarro G2121-i Cavity Ring-Down Spectroscopy δ^13^C stable isotope analyzer with an A0502 ambient CO_2_ interface, an A0201 Combustion Module, and an A0301 gas interface (CM-CRDS)^21^. To confirm the analyzer’s calibration, we ran a secondary standard of verified δ^13^C value (sucrose) every ten samples. All ^13^C concentrations are expressed in δ^13^C_VPDB_.

### Statistics and reproducibility

To study the allocation of labeled leucine into the different body tissues of workers and larvae (experiment 1), we used generalized linear mixed models followed by post-hoc pairwise comparisons (Tukey adjusted) to compare the δ^13^C concentrations between treatments. The models included the treatment, the type of body tissue analyzed, and their interaction as fixed factors, and the replicates were nested in the colony of origin as a random factor. The δ^13^C values were log_10_-transformed (x-minimum value+1) to fit a normal distribution.

To study the allocation of the labeled leucine from nectar and prey into the larval secretions (experiment 2), as the data did not follow a normal distribution even after transformation, we used a Kruskal-Wallis rank sum test followed by a Dunnett’s multiple comparison with control on the non-transformed data. The allocation of the two different labeled leucine into each body tissue of workers and larvae was analyzed using generalized linear mixed models, followed by a Dunnett’s multiple comparisons with control for each tissue. The replicates were nested in the colony of origin as a random factor, and the δ^13^C values were log_10_-transformed (x-minimum value+1) to fit a normal distribution.

To assess the role of the nectar in the colony’s survival (experiment 3), generalized linear mixed models with Poisson distributions followed by post-hoc pairwise comparisons (Tukey adjusted) were used to compare the number of dead workers and larvae, separately, between treatments, with the colony of origin as a random factor.

Statistical tests were run, and graphics were generated on R 4.0.3^55^. Statistical *p*-value was considered significant under 0.05.

### Reporting Summary

Further information on research design is available in the Nature Research Reporting Summary linked to this article.

## Supplementary information

Reporting Summary-New

## Data Availability

All the data used in this study are available via 10.5281/zenodo.7135100^56^.
